# Preparation and Structural Characterization of Complex Oxide Eutectic Precursors from Polymer–Salt Xerogels Obtained by Microwave-Assisted Drying

**DOI:** 10.3390/ma13081808

**Published:** 2020-04-11

**Authors:** Maria Vartanyan, Ilya Voytovich, Irina Gorbunova, Nikolay Makarov

**Affiliations:** 1 Department of Chemical Technology of Ceramics and Refractories, D.Mendeleev University of Chemical Technology of Russia, 125047 Moscow, Russia; lepro2020@mail.ru (I.V.); nikmak-ivmt@mail.ru (N.M.); 2 Department of Polymers Processing Technology, D.Mendeleev University of Chemical Technology of Russia, 125047 Moscow, Russia; giy161@yandex.ru

**Keywords:** sol–gel process, polyvinyl alcohol, carboxymethylcellulose, polyvinylpyrrolidone, ternary oxide eutectics, microwave-assisted drying, phase composition

## Abstract

Sol–gel synthesis is an acknowledged method for obtaining fine inorganic powders of a different nature. Implementation of water-soluble polymers as gel-forming media makes this technique even more readily available, especially in cases where conventional gel formation is suppressed. In polymer–salt solutions, polymers serve as scaffolds for salt constituents’ bulk crystallization. When dried, solid salt particles are deposited on the polymer surface or in polymer matrix pores, which leads to higher grain size uniformity. The present work discusses the effect of drying conditions on phase composition and structure characteristics of complex oxide eutectics in ternary systems, CaO–Al_2_O_3_–Y_2_O_3_ (CAY) and MgO–Al_2_O_3_–Y_2_O_3_ (MAY), obtained from polymer–salt compositions based on polyvinyl alcohol (PVA), Na-salt of carboxymethylcellulose (Na-CMC) or polyvinylpyrrolidone (PVP). Microwave-assisted drying proved to be more efficient compared to convective process; however such technique requires careful selection of gel-forming polymer.

## 1. Introduction

Sol–gel synthesis is a versatile and technologically viable method for obtaining ultra-fine and uniform powders of inorganic compounds of oxide [1] and non-oxide [2] nature. The sol–gel process typically uses soluble salts as precursors and comprises the following steps:

1. Dissolution of starting components in an appropriate media (generally water; however, the use of non-aqueous solvents [3] and supercritical fluids [4] has been reported) and sol formation;
2. Solvent evaporation and formation of gel structure;
3. Gel drying into xerogel;
4. Xerogel calcination to obtain the desired composition.

In recent years, a number of comprehensive studies on the advantages and limits of sol–gel synthesis for various applications have been published [5,6].

Typically, different sol–gel techniques vary by the starting materials and chemistry of the process. Along with conventional colloidal gels that are formed from metal oxide or hydroxide sols, gels are obtained by the following processes:

I. Alkoxide (metal–alcohol compounds Me(OR)_n_) hydrolysis. The primary reactions here are hydrolysis that can be described by the following equations:

Me(OR)_n_ + H_2_O = HO–Me(OR)_n-1_ + ROH; (1)

Me(OR)_n_ + nH_2_O = Me(OH)_n_ + nROH; (2)

or condensation according to the following scheme:

Me(OR)_n_ + H_2_O = HO–Me(OR)_n-1_ + ROH; (3)

(OR)_n-1_Me–OR + HO–Me(OR)_n-1_ = (OR)_n-1_Me–O–Me(OR)_n-1_ + ROH; (4)

(OR)_n-1_Me–OH + HO–Me(OR)_n-1_ = (OR)_n-1_Me–O–Me(OR)_n-1_ + H_2_O. (5)

Condensation leads to larger metal-containing molecules by the process of polymerization. Mixed metal alkoxides can also be used, and this mixture allows for the production of multicomponent oxides [7,8]. This technique holds many advantages in terms of product purity, adjustable batch and phase composition of powders and particle morphology and is closely linked to the metal–oxane polymer gel process [8].

II. Pechini method. This method was originally suggested in 1967 for obtaining dielectric materials and fine films based on lead titanates and niobates [9] and was later adjusted for producing ultra-fine powders of multicomponent oxides [10]. This technique exploits α-hydroxocarbon acid’s ability to form stable chelate complexes with metal ions and to etherify polyols. Generally, citric acid (HOOCCH_2_C(OH)(COOH)CH_2_COOH, C_6_H_8_O_7_) and ethylene glycol (HOC_2_H_4_OH) are used. A key feature of this method is that the gel network is formed by chemical interaction between organic components in which metal ions are captured.

III. Synthesis in polymer–salt systems. This technique supports the same concept as the Pechini method, and the gel-forming media here are water-soluble polymers, primarily polyvinyl alcohol (PVA), polyvinylpyrrolidone (PVP) and methylcellulose [11,12,13,14,15]. Sol–gel synthesis based on polymer–salt solutions has been successfully implemented to obtain nanoscale powders and thin films of various inorganic compounds, especially those with complex metal anions (i.e., tungstates, molybdates and manganates [11,12]). Synthesis of complex oxide compounds (i.e., cordierite [13], aluminomagnesian spinel [14] and lanthanum chromite [15]) has also been reported.

The main advantage of the polymer–salt sol–gel (PSSG) method is that it allows for controlling and precluding chemical interaction between otherwise incompatible inorganic precursors; however, it still raises several challenging issues to address. Firstly, this technique requires a fast way of drying gels to avoid crystal growth in solutions. Practitioners reported using spray-drying [11] or microwave-assisted drying [13,14]; microwave exposure holds much promise as a short-term treatment for such systems [16]. Secondly, a careful choice of gel-forming polymers is necessary. Polymers should maintain stability in solutions with high ionic power and readily decompose when heated, with minimal to no carbon residue to avoid product contamination.

Here, we report the PSSG synthesis and the physico-chemical properties of batch compositions in two ternary systems, CaO–Al_2_O_3_–Y_2_O_3_ and MgO–Al_2_O_3_–Y_2_O_3_, obtained with microwave-assisted drying, as suggested in [13,14,15], for further use as sintering aids in silicon carbide ceramics. Here, the effect of different ionic composition was studied, and different water-soluble polymers were employed, namely fully saponated PVA, high-weight PVP and Na-salt of carboxymethylcellulose (Na-CMC). These polymers are characterized by different unit cell structures (i.e., linear and cyclic) and different acid–base behavior, as these properties may affect the gel-formation process in the presence of inorganic ions.

The samples’ properties were characterized by means of a set of techniques (X-ray powder diffraction (XRD), FT-IR spectroscopy (FTIR), scanning electron microscopy (SEM) and optical microscopy) to better understand the state of polymer scaffolds in xerogels and to adjust xerogel formation parameters.

## 2. Materials and Methods

All inorganic salts (CaCl_2_·nH_2_O, Ca(NO_3_)_2_·nH_2_O, MgCl_2_·6H_2_O, Mg(NO_3_)_2_·6H_2_O, YCl_3_·6H_2_O, Y(NO_3_)_3_·6H_2_O, AlCl_3_·6H_2_O and Al(NO_3_)_3_·6H_2_O) were reagent grade from Sigma-Aldrich (Milan, Italy). Eutectic compositions and respective melting points are presented in Table 1, and the characteristics of the polymers used are shown in Table 2.

X-Ray diffraction patterns were obtained with a DRON-3M (Bourevestnik, Russia) Diffractometer operating at 40 kV and 10 mA and equipped with Cu Kα radiation (rotational rate 2 °/min). Absolute d-space error comprised ± 0.5%. Phases were identified with the Powder Diffraction File ASTM inorganic database [18].

IR transition spectra were acquired using a Nicolet IS10 Spectrometer (Thermo Fisher Scientific, Waltham, MA USA) (wavelengths range 400–4000 cm^−1^) from thin films, as well as powder samples pelletized with KBr. Characteristic bands were determined according to AIST: Spectral Database for Organic Compounds SDBS [19].

The microstructure of xerogels was studied with a VEGA3 Scanning Electron Microscope (TESCAN, Brno-Kohoutovice, Czech Republic) equipped with an INCA Energy 300 energy dispersive spectrometer (OXFORD Instruments, Abingdon, UK). Magnification power was ×5000 at an accelerating voltage of 10 kV.

Optical images were made with a MSFU-K Microscope-Spectrophotomer (LOMO, Russia) on dried polymer films.

**Figure 1:**
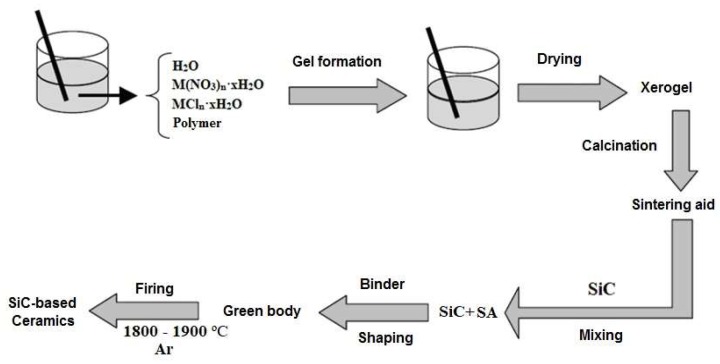
Xerogel formation and processing route.

Separate inorganic and organic samples were prepared by drying respective aqueous solutions in a laboratory microwave system (Discover BenchMate, CEM Corp., Matthews, NC USA) @ 2455 MHz to constant mass. Oxide eutectic compositions were prepared from polymer–salt solutions as discussed in [13,15]. Proper amounts of inorganic salts, calculated from ignition loss, were dissolved in 10% wt. aqueous polymer solutions and cured for 24 h at room temperature to obtain homogenous distribution of organic and inorganic components. In the next stage, gels were dried in a laboratory microwave system (Discover BenchMate, CEM Corp., Matthews, NC USA) @ 2455 MHz to constant mass. Resulting xerogels were hand-crushed in an agate mortar to pass through a 230-mesh sieve, and their phase composition and morphology were studied. For further use as sintering aids for SiC-based ceramics, the xerogels were subjected to heat treatment in air to burn out polymer remnants and complete eutectic phase formation. Figure 1 presents the PSSG synthesis scheme for sintering aids and their processing into SiC-based ceramic materials.

The sensitivity of inorganic constituents towards microwave (MW) drying was determined as sample dryness on a precise moisture analyzer AGS (AXIS, Gdansk, Poland) at a constant temperature of 300 °C. Dryness was calculated as follows:

w*_i_* = (M_0_ − M*_i_*)/M_0_, (6)

where M_0_ is initial sample weight and M*_i_* issample weight at *i* take.

## 3. Results and Discussion

### 3.1. Sensitivity of Initial Components Towards Microwave Exposure

Taking into consideration the complex nature of effects induced by MW radiation on aqueous systems, the first task of the study was to estimate the constituents’ stability. Organic and inorganic components were dissolved in freshly made bidistillate water and treated separately.

**Figure 2:**
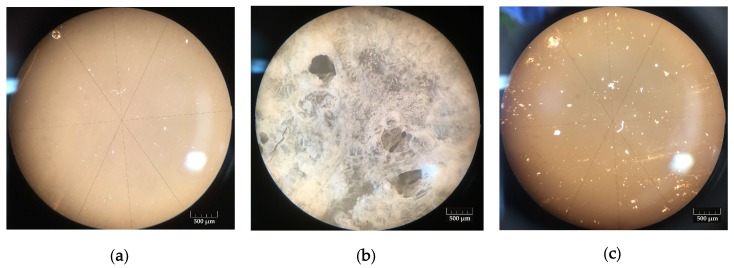
Optical images of (**a**) polyvinyl alcohol (PVA), (**b**) Na-salt of carboxymethylcellulose (Na-CMC), and (**c**) polyvinylpyrrolidone (PVP) dried films.

**Figure 3:**
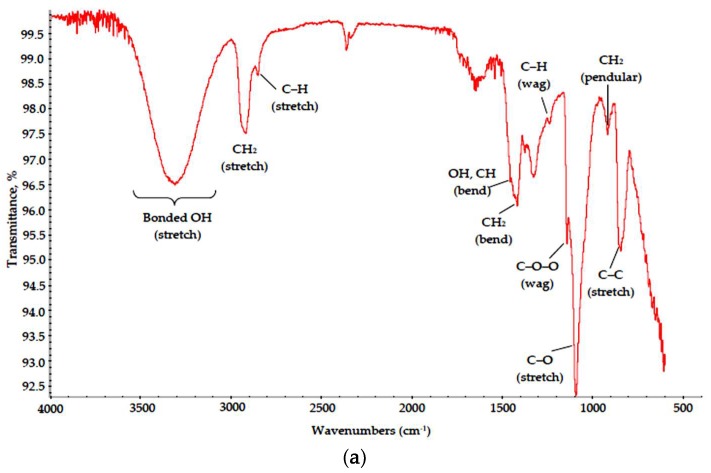

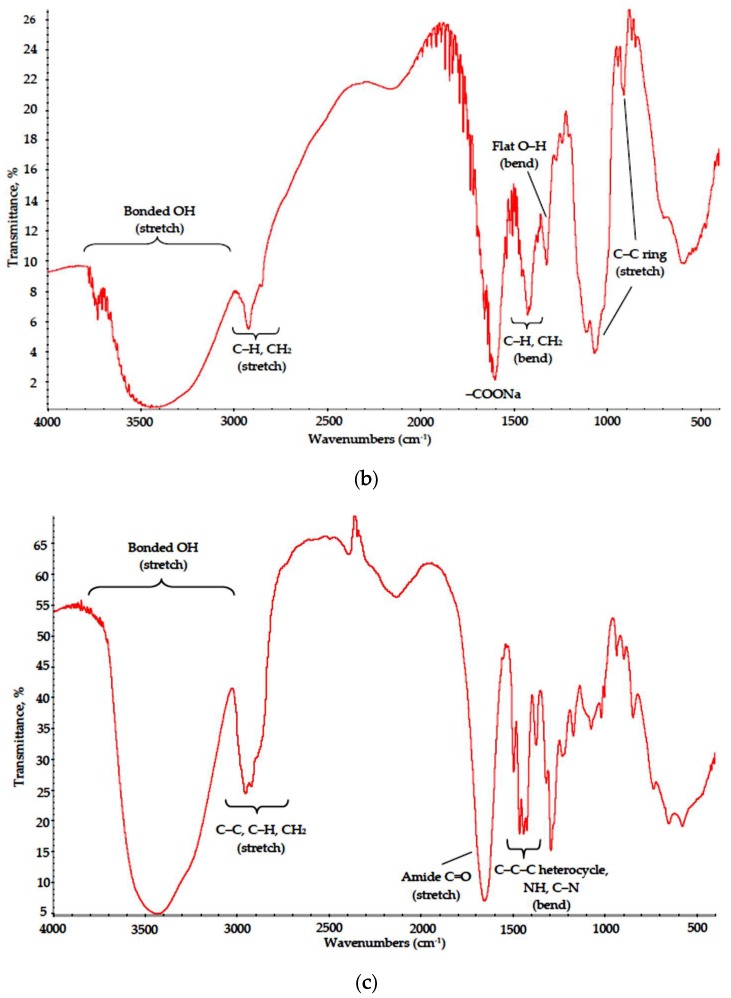
IR spectra of (**a**) PVA, (**b**) Na-CMC and (**c**) PVP dried films.

The stability of polymers was estimated by optical microscopy of thin films and FTIR data acquired from powders by comparison with standard curves; the respective data for PVA, Na-CMC and PVP are shown in Figure 2 and Figure 3 (see SBDS Nos. 2708, 6013 and 10479 for reference).

The presented data suggest that all polymers were almost unaffected by MW radiation. Characteristic bands corresponding to main structural elements were visible in all spectra. In the PVA spectrum, absorption bands associated with hydrocarbon backbone (a strong peak for C–C stretch at 850 cm^−1^ and a weak one for CH_2_ pendular oscillations at 900–930 cm^−1^, medium peaks for C–H wagging and CH_2_ bending modes at 1235 and 1430 cm^−1^, respectively, and strong peaks for C–H and CH_2_ stretch modes at 2840 and 2980–2900 cm^−1^, respectively) can be found, as well as those for OH groups (a peak for OH bending coupled with C–H bending at 1446 cm^−1^ and broad band at 3650–3000 cm^−1^ for bonded OH groups) and for C–O bonds (1050–1150 cm^−1^ for C–O stretch, 1140 cm^−1^ for C–O–O wagging modes). A small split band at 2390–2290 cm^−1^ can be attributed to captured CO_2_ admixtures [20].

In the Na-CMC spectrum, characteristic adsorption bonds also matched those reported in the literature [20]. A broad asymmetric peak at 3000–3650 cm^−1^ is typical for bonded OH groups in substituted celluloses; a weak split peak remains for CH and CH_2_ stretch modes at 2950–2800 cm^−1^, respectively. A broad peak at 1430–1370 cm^−1^ embraces CH and CH_2_ bending modes, peaks at 1110 and 895 cm^−1^ refer to C–C ring stretch and a small peak at 1350–1310 cm^−1^ corresponds to flat OH bending modes. A strong peak at 1600–1590 cm^−1^ is attributed to –COONa group [21].

The PVP spectrum also matched well with reference data [22]. A broad band at 3700–3000 cm^−1^ with maximum at 3450 cm^−1^ can be attributed to bonded O-H group stretch suggesting a high degree of hydrogen bonding. A split band at 3000–2850 cm^−1^ corresponds to CH_2_, C–C and C–H stretch, a strong peak at 1650 cm^−1^ reflects C=O stretch in cyclic amides and medium intensity bands in 1480–1420 cm^−1^ range correspond to bending modes of C–C–C heterocycle as well as those of NH and C–N in cyclic amides.

Optical microscopy of the polymer films obtained by MW drying of 10% wt. aqueous solutions of the studied polymers proved that no signs of sample destruction were observed. All films had dense structure; PVP and PVA films were optically transparent. Because MW drying is a short-term and highly non-equilibrium process, we managed to obtain an amorphous PVA film without any visible crystallization.

**Figure 4:**
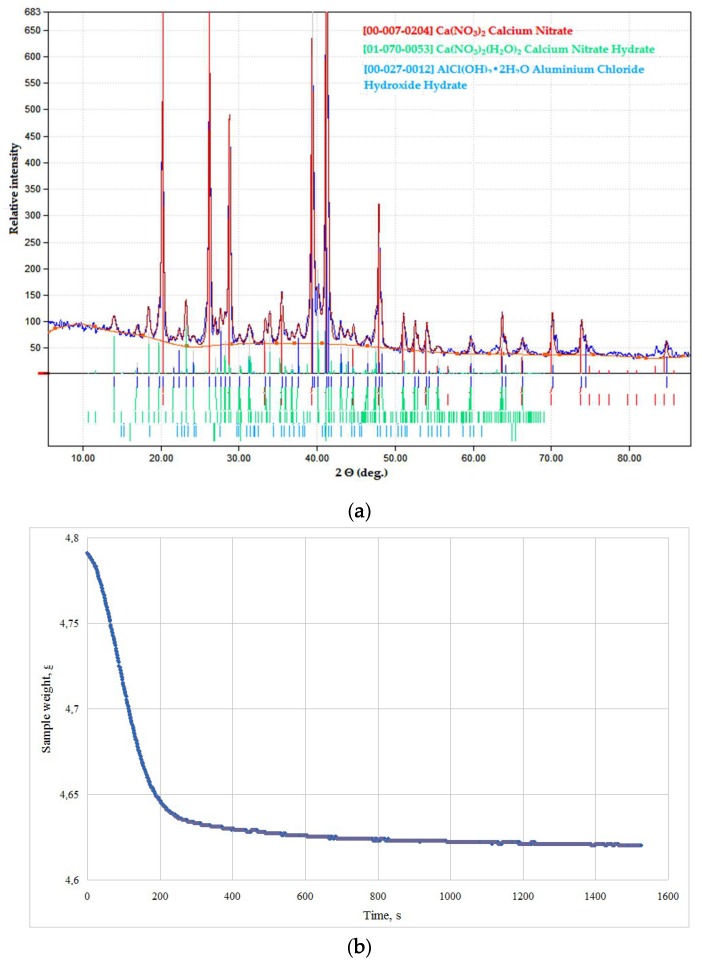
Products of a nitrate solution microwave (MW) drying in the CaO–Al_2_O_3_–Y_2_O_3_ (CAY) system: (**a**) phase composition; (**b**) weight loss.

Initial salt mixtures were more sensitive towards MW exposure. Dried products in the CaO–Al_2_O_3_–Y_2_O_3_ (CAY) and MgO–Al_2_O_3_–Y_2_O_3_ (MAY) systems were prepared with chlorides and nitrates of respective salts. According to XRD data, stable anhydrous salts were formed in both systems, and the samples remained much as they were, even for thermally instable nitrate compositions (see Figure 4). Dryness measurements also proved anhydrous salt formation and experimental weight loss of 3.5–3.7% and 0.7–1.2 % wt. for the CAY and MAY powders, respectively, corresponding to desorption of water and possible secondary calcium salt dehydration.

### 3.2. Polymer–Salt Systems

**Figure 5:**
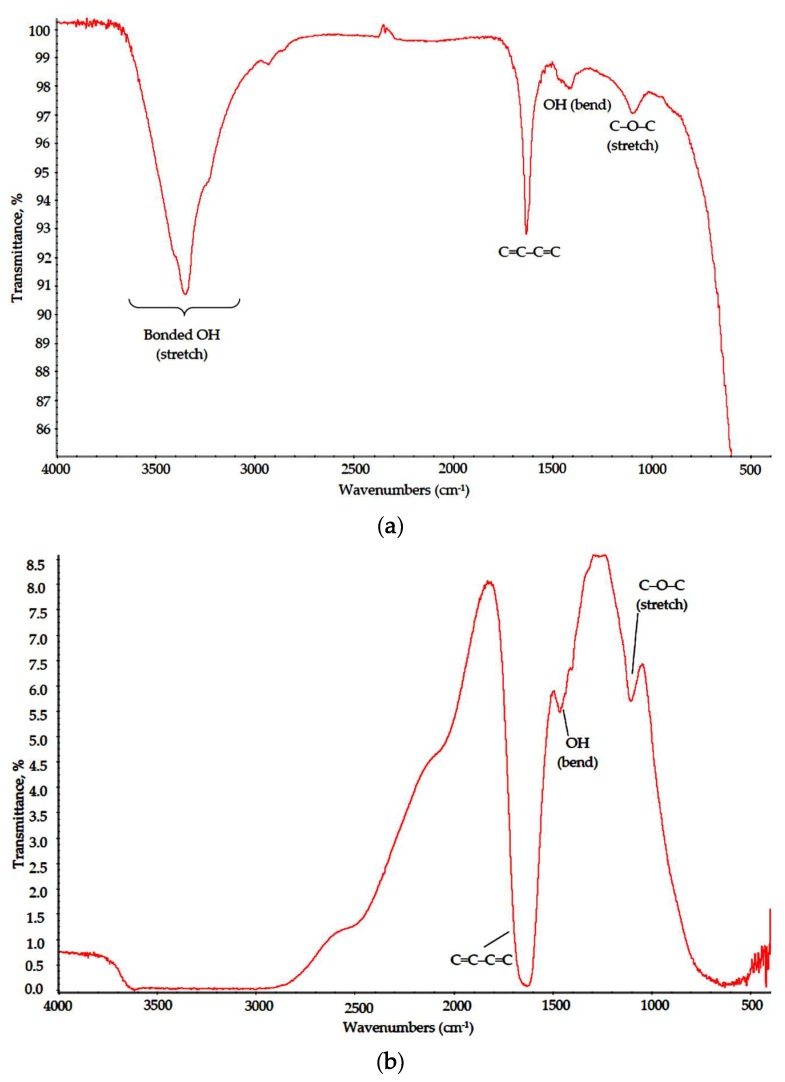
IR spectra of (**a**) PVA–CAY and (**b**) PVA–MAY powders.

Polymer–salt systems were prepared with mixed anion inorganic constituent (CaCl_2_ + AlCl_3_ + Y(NO_3_)_3_) to promote the thermal destruction of polymer scaffolds in final-stage calcination. Quite unexpectedly, under MW treatment, the polymer–salt systems, unlike pure solutions, underwent drastic structural changes. Polymers in both Na-CMC and specifically PVA-based xerogels demonstrated signs of oxidation and, in the case of Na-CMC, the initial stage of fiber carbonization. In the FTIR spectra of PVA–CAY (Figure 5), the “fingerprint” area (1500–700 cm^−1^) contained almost no peaks associated with carbohydrate moieties (CH, CH_2_). Observed absorption bands can be attributed to bonded OH group stretch (maximum at 3450–3400 cm^−1^); a strong bond at 1640–1600 cm^−1^ refers to conjugated alkene structures (C=C–C=C); this peak appears in the Na-CMC spectrum as well (discussed below). To distinguish this peak from nitrate (-O-NO_2_) stretch, we prepared PVA–MAY samples with a chloride-only batch (MgCl_2_ + AlCl_3_ + YCl_3_), and the same strong peak was observed in this range. Minor peaks at 1420 and 1110 cm^−1^ can be attributed to OH bending and C–O–C stretch, respectively.

**Figure 6:**
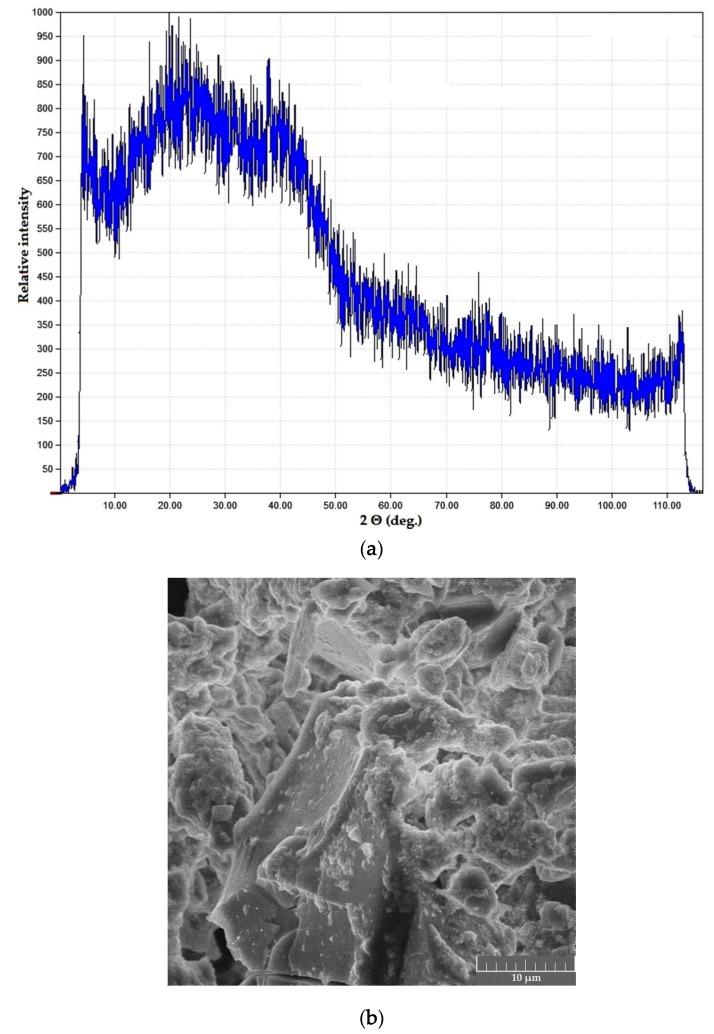
Dried products in the PVA–CAY system: (**a**) phase composition; (**b**) microstructure.

Powder samples in both systems were black in color, with ultra-fine powder precipitates (mean particle size below 1 μm) and carbon deposits visible in SEM images; XRD curves of the samples demonstrated a characteristic halo effect (Figure 6).

Such poor performance of PVA-based compositions under MW treatment may be explained as a result of two subsequent processes. PVA in water solutions behaves as a weak polyanionic electrolyte, and in the curing stage, the presence of polyvalent cations (Ca^2+^, Mg^2+^, Al^3+^, Y^3+^) promotes the formation of coordination complexes; such effect has been reported for transition metals in [23]. This results in brine acidification; therefore, in the drying stage, large polymer structures are subjected to heating in diluted acidic media. This leads to chain decomposition and to a certain extent, oxidation; still, the drying process is performed too fast for significant polymer backbone destruction.

**Scheme 1:**
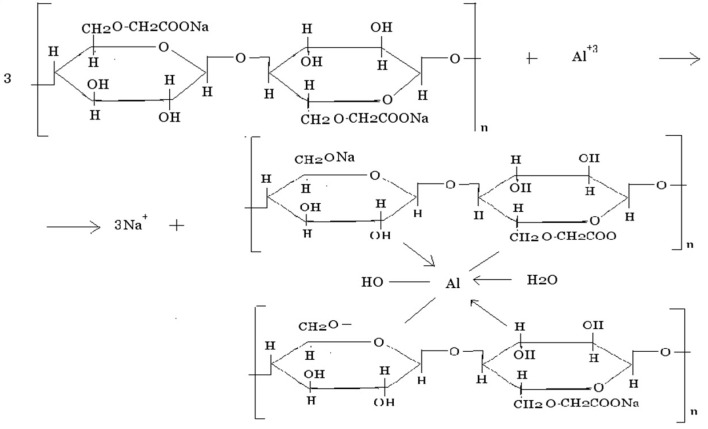
Cross-linking of Na-CMC chains in presence of Al^3+^ ions.

**Figure 7:**
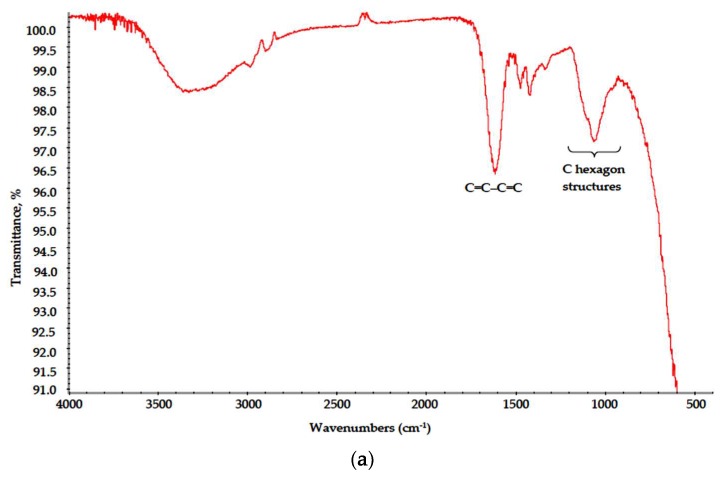

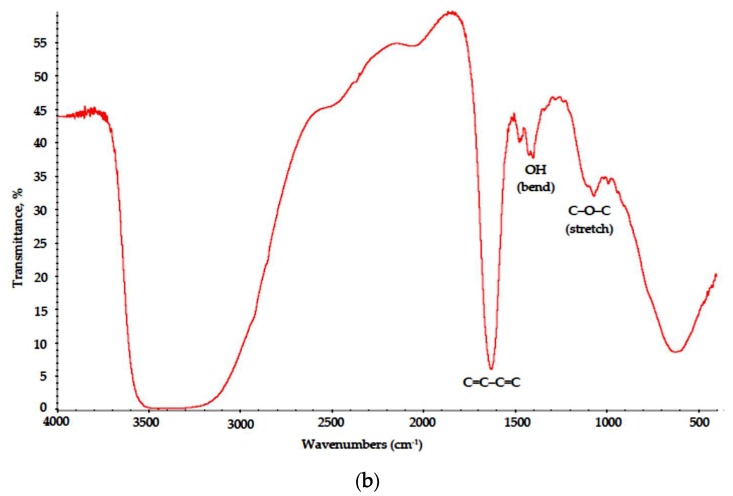
IR spectra of the (**a**) Na-CMC–CAY and (**b**) Na-CMC–MAY powders.

The same behavior is exhibited by Na-CMC, which is a typical polyelectrolyte. In the presence of polyvalent cations, polymer chains are prone to cross-linking; a model reaction for Al^3+^ may be presented as follows (Scheme 1):

This results in cation exchange and polymer precipitation from the brine. In the drying stage, linked cellulose fibers are not only locally overheated due to MW radiation absorption, but are also subjected to intense heating in aqueous solution of thermally unstable oxidant NaNO_3_; thus, surface pyrolysis is initiated, and heteroaromatic polycondensed ribbon with distinct regular carbon hexagons (chicken-wire structures) is formed, as suggested in [24]. In the FTIR spectra of the Na-CMC–CAY and Na-CMC–MAY powders (Figure 7), these structures are described with broad medium intensity absorption bands at 1200–900 cm^−1^ [25] and a strong peak at 1640–1600 cm^−1^, as discussed above. In purely chlorine solution (Na-CMC–MAY powders), the oxidation effect is less pronounced; however, conjugated alkene structures (C=C–C=C) are still formed.

**Figure 8:**
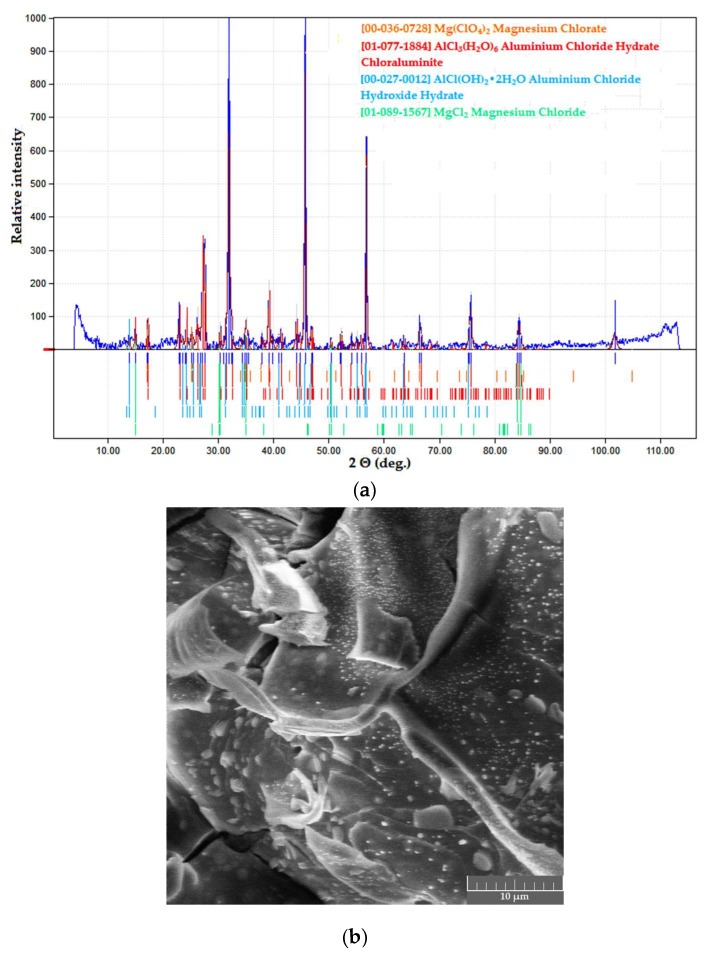
Dried products in the Na-CMC–MAY system: (**a**) phase composition; (**b**) microstructure.

Powder samples in both systems were also black in color, yet their microstructure and phase composition were different from those with PVA. Cellulose fibers served as an activated carbon substrate on which salt particles precipitated and grew into well-crystallized structures (mean particle size 3–4 μm) as presented in the XRD patterns (Figure 8).

**Figure 9:**
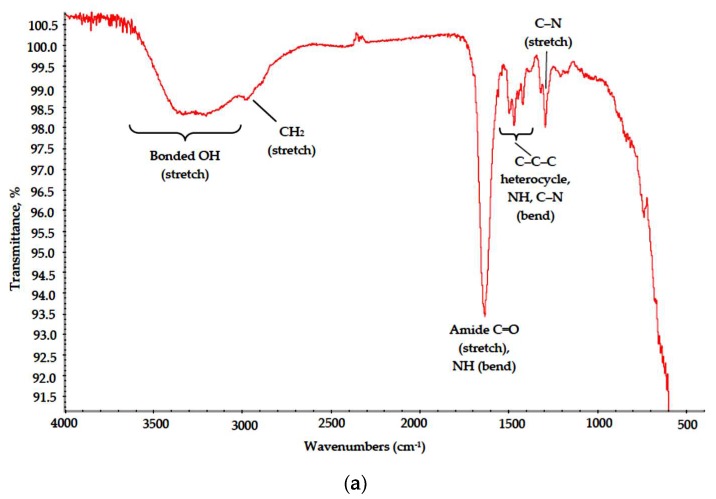

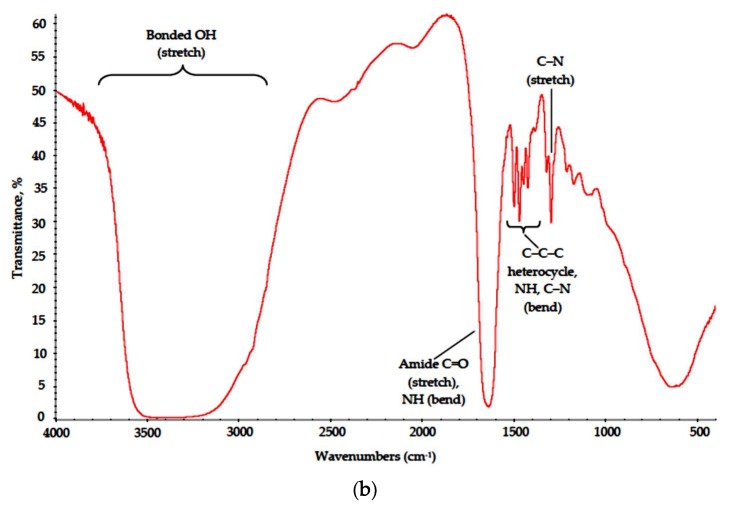
IR spectra of the (**a**) PVP–CAY and (**b**) PVP–MAY powders.

The PVP-based samples were less subjected to destruction. This outcome is supported by FTIR data (Figure 9) and SEM results. Major absorption bands in the spectra of both PVP–CAY and PVP–MAY corresponding to carbohydrate moieties remained. Low-intensity bonds attributed to CH_2_ stretch can be found at 2900 cm^−1^ in both spectra, even though for PVP–MAY, peaks in the high wavenumber range were masked with an extremely strong and broad band at 3700–2700 cm^−1^ for bonded OH groups.

**Scheme 2:**
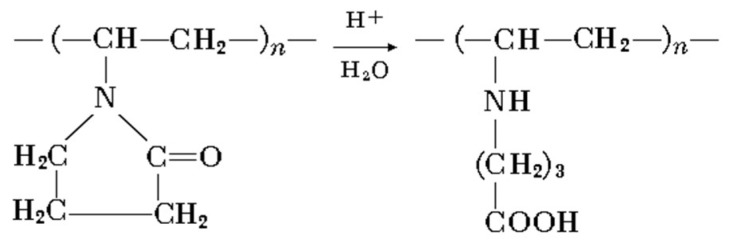
Lactam ring hydrolytic splitting [26].

A minor shift of C=O stretch peak to 1640 cm^−1^ may be attributed to lactam ring hydrolytic splitting, according to Scheme 2 ([26]):

Where poly-N-vinyl-γ-aminobutyric acid is formed. NH and C–N groups appear with their respective peaks at 1650 (secondary NH bending) and 1220 cm^−1^ (C–N stretch).

Absorption bands corresponding to bending modes of C–C–C heterocycle as well as those of NH and C–N in cyclic amides retained their position in the range of 1480–1420 cm^−1^.

**Figure 10:**
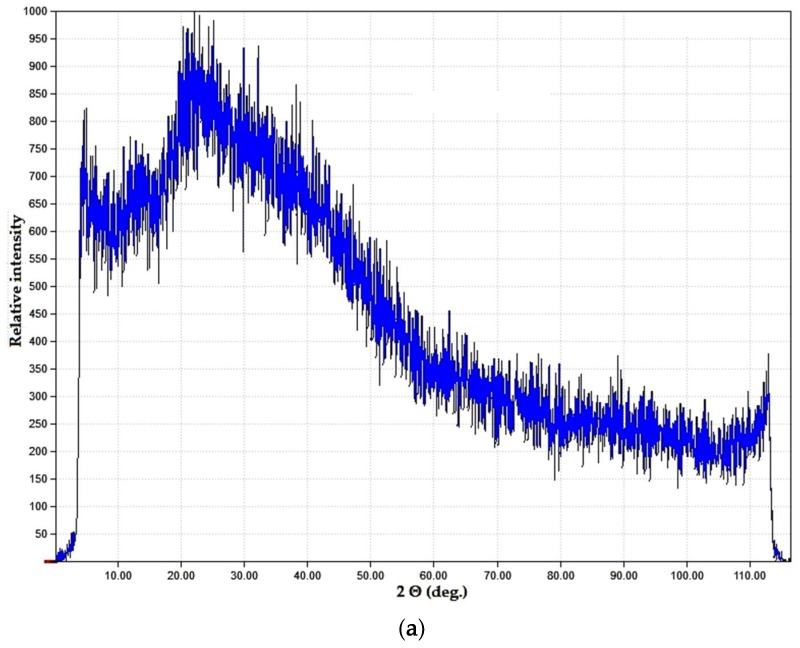

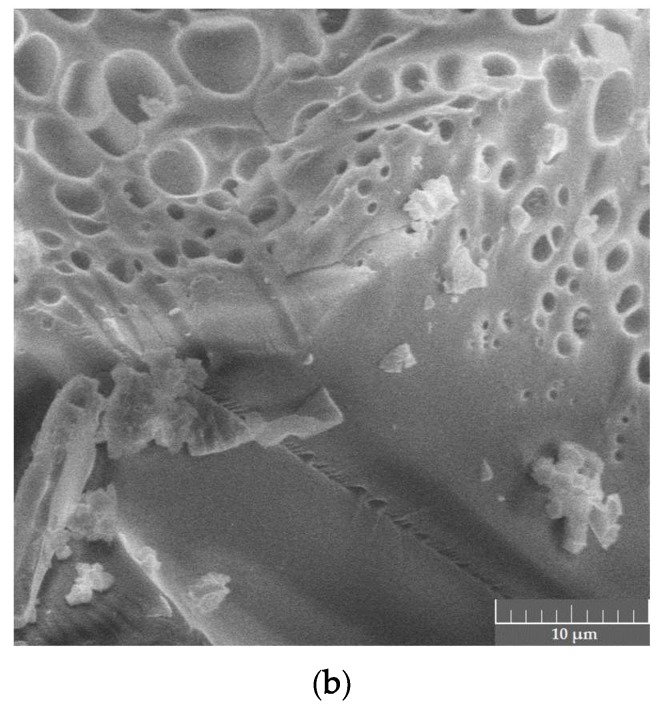
Dried products in the PVP–CAY system: (**a**) phase composition; (**b**) microstructure.

Spectral data were in good correlation with phase microstructure studies as well as visual evaluation of the samples. Both the PVP–CAY and PVP–MAY powders were light- colored with a slight yellowish tone. In micrographs, a well-set polymer structure is visible with no presence of carbon deposits; spherical pores may result from water evaporation during MW drying. Salts precipitated primarily on structural defects as particle aggregates (mean size 1.5–2 μm) forming a halo in the XRD pattern (Figure 10).

Such vivid difference in the studied polymers’ behavior, especially between the two with vinyl moiety (PVA and PVP), obviously results from their different functionalization and corresponding oxidative stability. In PVA and Na-CMC, the functional groups available for interaction in energy-intensive processes are OH-groups, and they are easily removed even in weak oxidative conditions (such as boiling in diluted mineral acids)with possible polymer chain destruction. Even though all polymers are capable of complex formation with metal cations, cyclic amide (lactam) moiety in PVP is a far more intricate structural element that can undergo a series of chemical transformations starting with hydrolytic ring splitting. Increased molecular weight of PVP also suppresses oxidation due to steric constraints. This improves the oxidative stability of the polymer scaffold and precludes the contamination of the inorganic constituent with carbon, which occurs in Na-CMC- and PVA-based xerogels.

## 4. Conclusions

Polymer–salt solutions hold much promise as media for synthesis of complex oxide batches with pre-defined phase composition and particle morphology. The use of short-term, highly non-equilibrium drying techniques, such as microwave-assisted drying, makes it possible to obtain ultra-fine inorganic precursor batches that may easily be processed into a target product.

The experiment proved that in the absence of inorganic (salt) constituent, microwave exposure had no particular effect on the structure of all the studied polymers. Likewise, in the absence of organic molecules, salt crystallohydrate batches transformed into stable anhydrous salts without further decomposition. However, when combined into a polymer–salt system, a considerable decay of organic constituents took place with no regard to cation type and oxidative ability of anions (chlorides or nitrates). The polymer decomposition process was rapid and strong in carboxymethylcellulose and especially in polyvinyl alcohol, an outcome that may result from both their simple functionalization (reactive substituents in both polymers are aliphatic OH-groups) and their ability to form insoluble salts or stable metal–polymer complexes, respectively; this in turn leads to the formation of large reaction sites subjected to local overheating and subsequent thermo-chemical polymer chain destruction.

Even though polyvinylpyrrolidone exhibits complex-forming ability on par with that of polyvinyl alcohol, this polymer demonstrated higher oxidative stability under microwave exposure. A possible reason for this may be a combination of high molecular weight and a complex lactam substituent in a unit cell that acts as a protective element for the polymer backbone, as in aggressive media, it may enter a series of chemical transformations. Moreover, the presence of a N–C=O group capable of electron density redistribution improves the stability of condensed matter located in the microwave frequency electromagnetic field.

Phase composition and structure analysis of xerogels showed that under microwave exposure, the use of non-fibrous, non-ionogenic scaffolds (i.e., those with vinyl moiety) resulted in the decomposition of initial salts and the formation of ultra-fine amorphous powders. The quality of these powders strongly depended on polymer stability; carbon residue from polymer destruction readily formed intercalates with oxide compounds. The fibrous polymer in these conditions served as an activated carbon substrate on which salt crystals formed and grew into relatively large particles with well-defined structure.

Based on the present research, a most suitable candidate polymer for polymer–salt synthesis should exclude polyelectrolytes, preferably comprise a linear chain and complex substituent in a unit cell and fully decompose when calcined at temperatures below that of salts’ thermal decomposition. Further studies may be aimed at polyvinylpyrrolidone of lower molecular weight as well as polyacrylate derivatives.

## Figures and Tables

**Table 1:** Compositions (% wt.) and melting points of eutectic batches (°C) [17].

	Oxide	CaO	Al_2_O_3_	Y_2_O_3_	MgO	Melting Point, °C	Eutectic Phases
Eutectic System							
CaO–Al_2_O_3_–Y_2_O_3_ (CAY)		32.0	37.0	31.0	—	1675	2CaO·Y_2_O_3_·Al_2_O_3_, CaO·Al_2_O_3_, 3Y_2_O_3_·5Al_2_O_3_
MgO–Al_2_O_3_–Y_2_O_3_ (MAY)		—	43.0	50.9	6.1	1775	Al_2_O_3_, MgO·Al_2_O_3_, 3Y_2_O_3_·5Al_2_O_3_

**Table 2:** Structural formulae and characteristics of polymers.

Name	Unit Cell Formula	Grade	Regulatory Document (in Russian)
PVA	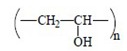	16/1	GOST 10799-78
Na-CMC	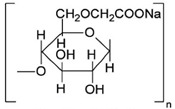	pharmaceutical	TU 2231-034-79249837-2006
PVP	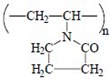	K-90	FSP 42-0345-4367-03
